# This is the place: a multi-level analysis of neighbourhood correlates of adolescent wellbeing

**DOI:** 10.1007/s00127-023-02531-y

**Published:** 2023-08-22

**Authors:** Jose Marquez, Neil Humphrey, Louise Black, Sophie Wozmirska

**Affiliations:** 1 University of Manchester, Manchester, UK; 2 The Co-Op. The Cooperative Group, Manchester, UK

**Keywords:** Adolescence, Wellbeing, Mental health, Neighbourhood, Epidemiology

## Abstract

**Objective:**

Adolescent wellbeing is a key research and policy priority, but little is known about neighbourhood-level influences. This study examined the extent to which adolescents’ life satisfaction and internalising symptoms vary between neighbourhoods, and which neighbourhood characteristics are associated with individual outcomes.

**Method:**

Baseline data from the #BeeWell cohort study in Greater Manchester (England) including 35,902 adolescents (aged 12–15) across 243 neighbourhoods were linked to neighbourhood characteristics (e.g. access to education and health services, leisure facilities) from the Co-op’s Community Wellbeing Index and analysed using multi-level regression.

**Results:**

Neighbourhoods explained 0.61% and 1.17% of the variation in life satisfaction and internalising symptoms, respectively. Socio-demographic inequalities in these outcomes varied across neighbourhoods. Several neighbourhood characteristics were associated with wellbeing, but differences across model specifications were observed (e.g. adjusted vs unadjusted; unique associations vs grouped domains). However, higher levels of perceived wellbeing support from local people were associated with lower internalising symptoms in all models. Other characteristics associated with better wellbeing outcomes in various models included lower GP antidepressant prescription rates, and better access to health services, areas for leisure, and good places to spend free time.

**Conclusion:**

Neighbourhoods account for a small but significant proportion of the variance in adolescent life satisfaction and internalising symptoms. Some neighbourhood characteristics (notably neighbourhood social capital) are associated with these outcomes at the individual level, and disparities in these outcomes for some groups vary across neighbourhoods. Our findings speak to the role of place as a determinant of adolescent wellbeing, with consequent implications for intervention.

**Supplementary Information:**

The online version contains supplementary material available at 10.1007/s00127-023-02531-y.

## Introduction

Improving our understanding of the determinants of wellbeing during adolescence is a key research and policy priority [1] for a number of reasons. First, subjective wellbeing starts to decline in the transition from childhood [2]; furthermore, the majority of lifetime cases of mental ill health begin in adolescence, with a peak age of onset of 14.5 years [3]. Second, poor wellbeing in adolescence is predictive of a range of maladaptive outcomes in later life [4]. Third, the prevalence of mental health difficulties among adolescents has increased significantly in recent years [5], while their life satisfaction has declined sharply [6]. In terms of determinants, it is generally accepted that adolescent wellbeing is influenced by a range of factors relating to the individual, the developmental contexts in which they reside (e.g. family, school, neighbourhood) [7, 8], and the interactions within and between them. Accordingly, a pluralistic and multi-level perspective is required [9]. This paper focuses on the role of ‘place’. Specifically, we examine the extent to which variability in adolescent wellbeing (operationalised here through two key indicators: internalising symptoms and life satisfaction) is attributable to differences between neighbourhoods, and which neighbourhood characteristics are associated with individual outcomes. Far less is known about the role of neighbourhoods than other developmental contexts such as family and school [7, 10]. As such, an analysis of this kind offers an important and timely contribution to the epidemiology of adolescent wellbeing, especially given the fact that intervention often happens at this level [11].

In considering what we know from existing research, a useful starting point is the proportion of variance in children and adolescent wellbeing that might be attributable to differences between neighbourhoods, expressed as the intra-cluster correlation coefficient (ICC). An early review of neighbourhood effects on children and adolescent health and wellbeing provided clear evidence that this varies by outcome and other methodological and analytic factors, but is typically in the 1–5% range [12]. In a more recent illustrative example, Aminzadeh et al.’s analysis of 5567 adolescents nested in 262 neighbourhoods in New Zealand reported an ICC of 1% for self-reported wellbeing [13]. By contrast, Dunn et al.’s study of 16,172 adolescents nested in 2118 neighbourhoods revealed an ICC of 3% for depressive symptoms [14]. Thus, a small but significant proportion of the variability in adolescent wellbeing is attributable to differences between neighbourhoods. There is limited comparable evidence in relation to adult wellbeing, though that which does exist indicates somewhat larger ICCs [15–17].

However, assessment of the influence of neighbourhoods on adolescent wellbeing should not be solely based on ICC values as we know that low ICCs can coexist with important fixed effects of contextual variables [18]. A range of neighbourhood characteristics have been shown to be associated with adolescent wellbeing generally and/or life satisfaction or internalising symptoms specifically, including for example exposure to violence [19], perceived safety [10], socio-economic deprivation [20], areas to play [21], autonomous spaces away from the gaze and direction of adults [22], green space quality and quantity [23], residential instability [24], and social capital [13], which includes aspects such as sense of belonging to the community [25] and neighbourhood support [26]. For example, in the aforementioned study by Aminzadeh et al. [13], neighbourhood social capital was found to be associated with adolescent wellbeing. More specifically, increased levels of social cohesion (i.e. cognitive social capital measured in terms of general perception of mutual trust, reciprocity, sense of community and safety) and levels of youth membership in community organisations (i.e. structural social capital) were associated with higher wellbeing (ES range 0.14 to 0.18) even after controlling for ethnicity, age, gender and socio-economic status.

However, our ability to make inferences from the above evidence about the role neighbourhoods play in adolescent wellbeing is currently limited by several issues, which collectively can lead to *underestimation* over *overestimation* of neighbourhood effects. First, many studies do not genuinely test neighbourhood effects because they fail to take a multi-level analytic approach (e.g. adolescents nested *within* neighbourhoods); for example, 30% of studies reported in Visser et al.’s recent systematic review of neighbourhood deprivation effects on young people’s wellbeing used single-level analyses [20]. Second, area effects studies tend to rely on administrative units of analysis (e.g. census tract data), as opposed to those constructed on the basis of how residents experience and define their neighbourhoods [27, 28]. This can cause two related issues: the modifiable areal unit problem (statistical bias arising from the fact that summary values such as population density are influenced by the shape and scale of the neighbourhood aggregation unit), and the uncertain geographic context problem (the effects of neighbourhood characteristics being affected by how neighbourhoods are geographically demarcated and the extent to which these areal units deviate from the “true causally relevant” geographic context [27, 29]. Third, reliance on participant-assessed neighbourhood exposures can lead to same source bias (e.g. the wellbeing of an individual may influence their perceptions of their neighbourhood) [30]. Fourth, much of the extant evidence base focuses on the effects of neighbourhood socio-economic deprivation [20, 25]. By contrast, less is known about other features and characteristics such as relational attributes (e.g. feelings of trust and support). Although this creates a challenge in as much as data on such attributes are likely to be participant-assessed (leading back to the same source bias issue noted above), this can be mitigated through collective assessment (i.e. aggregating multiple responses within the same neighbourhood) [30]. This yields a more valid measure of the objective neighbourhood characteristic of interest by averaging over measurement error in individual responses [18], while also facilitating a truly multi-level approach (and, thus, a genuine test of neighbourhood effects, thereby also addressing the first issue noted above).

In light of the above, the aims of the current study were to (a) examine the extent to which variability in adolescent internalising symptoms and life satisfaction is attributable to differences between neighbourhoods (including whether inequalities in wellbeing between distinct socio-demographic groups vary across neighbourhoods); and (b) which neighbourhood characteristics (e.g. access to health services, trust, green space) are associated with individual outcomes. In pursuit of these aims, we sought to contribute to the understanding of neighbourhood effects on adolescent wellbeing by leveraging secondary analysis of a unique, contemporary dataset (the #BeeWell study, described in the Method) linked to publicly available data on neighbourhood characteristics derived from the Co-op’s Community Wellbeing Index (CWI). More specifically, we were able to build upon and extend the evidence base by taking a multi-level approach to test neighbourhood effects; employing units of analysis constructed on the basis of how residents experience and define their neighbourhoods; using both participant-assessed and administrative data on neighbourhood characteristics; and examining the association between a range of said characteristics and individual wellbeing outcomes.

## Methods

### Study sample

We use cross-sectional individual-level survey data collected in the first wave of the #BeeWell project in Greater Manchester [31, 32], and linked CWI neighbourhood data [33, 34].

The overarching research aim of #BeeWell is to improve our understanding of the development and drivers of wellbeing in adolescence. The project uses a hybrid population cohort study design [8], including two main elements: (i) a truncated longitudinal study in which participants are followed annually from age 12–15 (i.e. from Year 8 to Year 9 to Year 10 of secondary school; Sample 1); and (ii) a cross-sectional study including data for participants aged 14–15 each year (i.e. those in Year 10 of secondary school at a given data point; Sample 2). Our analysis draws on the first annual wave of #BeeWell data collection (Autumn 2021), combining data from Samples 1 and 2, as these data were available at the point of analysis for the current study.

Individual-level data were collected on a wide range of wellbeing domains/indicators (e.g. life satisfaction) and drivers (e.g. sleep), as well as multiple individual characteristics (e.g. ethnicity) in a sample of 37, 978 adolescents. Analysis of the characteristics of this sample [35] indicate that it closely mirrors the composition of the principal population of interest, young people aged 11–16 in the Greater Manchester city-region, meaning that sample weights did not need to be applied. The #BeeWell sample also mirrors the composition of the 11–16 population nationally, though with a somewhat higher proportion of Asian (and lower proportion of White) young people than is seen across England [34].

Neighbourhood data (see description of CWI in Measures section below) from 2021 were linked to #BeeWell individual-level data via the participants’ residential postcodes. The final sample resulting from merging these data sets included 35,970 adolescents residing in 278 neighbourhoods in Greater Manchester. To ensure sufficient power in the analysis, neighbourhoods with fewer than 5 observations were removed (*n* = 35,902; 243 neighbourhoods) [36]. For the same reason, in the analysis of random effects (see Analytical Approach below), neighbourhoods with fewer than 10 observations were excluded (*n* = 35,820; 231 neighbourhoods).

### Measures

#### Individual-level measures

Wellbeing of young people was measured using data from the #BeeWell survey on life satisfaction and internalising symptoms. The former was assessed via the life satisfaction item from Office for National Statistics’ personal wellbeing item bank [37], which provides a score of 0–10, with higher scores indicating greater life satisfaction. The latter was assessed via the 10-item emotional difficulties subscale of the Me and My Feelings (M&MF) measure [38]. Each item is rated from 0 to 2, yielding a total score of 0–20, with higher scores indicating greater internalising symptoms. The M&MF measure was designed for use with children and young people aged 8 and above. Psychometric assessment has provided evidence of internal consistency, convergent and discriminant validity [39]. In our sample, the Cronbach’s alpha for this measure was 0.88.

Data on individual characteristics used as co-variates in our models were derived from two sources. First, linked administrative data on participants’ age, sex, ethnicity, and free school meal (FSM) eligibility were provided by the 10 Greater Manchester Local Authorities. Second, data on gender identity and sexual orientation were drawn from the #BeeWell survey.

#### Neighbourhood-level measures

Neighbourhood demarcation was drawn from the CWI. In brief, this is an online tool developed by the Co-operative Group to provide an index of community wellbeing, defined as “a collective feeling of leading a ‘good life’, shared and created by people and systems. Community wellbeing is more than people’s individual wellbeing; it is the relationship between people and place” [34]. CWI neighbourhoods are known as ‘seamless locales’, whose boundaries are designed to reflect how people familiar with a given area would divide them. The creation of these locales (hereafter, referred to as neighbourhoods) by the Co-operative Group (and their partners at Geolytix) involved a combination of user feedback, data on observed travel, work and shop patterns, administrative data (e.g. Land Registry House Price Paid data), and expert judgement [34]. The resulting geographic units were considered to be better suited to our analysis than those based solely on administrative data. As noted above, there were 278 such units initially represented in our sample. For context, this makes seamless locales *more* granular than middle super output areas, but *less* granular than lower super output areas (two commonly used administrative geographic units).

Neighbourhood-level factors were derived from two sources. First, updated 2021 CWI data were used, spanning a range of domains deemed to be relevant to young people’s wellbeing. These included education and learning (access to schools, school quality, and access to libraries); health (access to health services, GP prescription rates for antidepressants); economy, work and employment (unemployment, free school meals); culture, heritage and leisure (areas for leisure, distance to museums/art galleries/theatres); transport, mobility and connectivity (public transport); housing, space, and environment (public green space, public space, traffic pollution, and air quality); relationships and trust (social spaces, neighbourhood watch); and, equality (house price gap). Underpinning data for each indicator are drawn from a range of publicly available information from sources including, for example, the Office for National Statistics, the National Health Service, and the Electoral Commission. CWI variables are transformed for ease of interpretation so that they are on a scale of 0–1, with 1 always representing the optimal score for a given indicator (e.g. better access to schools, smaller house price gap) and 0 the worst. Further information about each measure is noted in Table 1; how each score is calculated and the source of each underpinning data item is available in the CWI technical report [33]. Second, #BeeWell local environment survey data items pertaining to feeling safe, support for wellbeing among local people, trust in local people, neighbour helpfulness, and good places to spend free time, were aggregated to the neighbourhood level. These items were adapted from the Health Behaviours in Schools checklist [32].

**Table 1:** Characteristics of the sample (35,902 adolescents, 273 schools) and descriptive information of study variables

CWI—community variables (from 0 to 1; no missing data)	Description	Range	Mean	SD	
Education and learning					
Access to schools	Derived from the total count of schools, and distance to the nearest state school	0.37–1.00	0.94	(0.06)	
School quality	Derived from the % of schools rated good or outstanding by Ofsted	0.01–0.99	0.61	(0.28)	
Access to libraries	Derived from the distance to the nearest library	0.33–1.00	0.90	(0.14)	
Health					
Access to health services	Derived from the distance to the nearest GP, hospital, mental health service, and pharmacy	0.80–1.00	0.96	(0.04)	
GP prescription rates for antidepressants	Derived from the % of antidepressants prescribed in relation to total patients within the neighbourhood. In the case of no GP within the Locale, the nearest two were selected	0.00–0.99	0.44	(0.28)	
Economy, work and employment					
Unemployment	Derived from the % of adults within the neighbourhood claiming job seekers allowance	0.01–0.98	0.13	(0.17)	
Free school meals	Derived from the proportion of children at schools within the Locale taking Free School Meals	0.01–1.00	0.21	(0.24)	
Culture, heritage and leisure					
Areas for leisure	Derived from the distance to the nearest leisure facility, grass pitch, sports hall, and swimming pool	0.74–1.00	0.93	(0.05)	
Museums, art galleries, music halls, theatres	Derived from the distance to the nearest museum, art gallery, and theatre	0.39–1.00	0.82	(0.12)	
Transport, mobility and connectivity					
Public transport	Derived from the count of bus stops per 10,000 population, distance to a major rail stations, and distance to any rail station	0.31–0.91	0.68	(0.08)	
Housing, space and environment					
Public green space	Derived from the % of the area of the neighbourhood made up of green space	0.00–0.99	0.86	(0.10)	
Public space	Derived from the distance to the nearest community centre, and the nearest playground	0.43–1.00	0.88	(0.12)	
Traffic pollution	Derived from the maximum count of both HGV and total traffic going through the neighbourhood	0.01–1.00	0.26	(0.24)	
Air quality	Derived from the count of certain gases in the air (NO2, SO2, PM10)	0.05–0.52	0.18	(0.11)	
Relationships and trust					
Social spaces	Derived from the distance to the nearest pub, café, community centre, and playground	0.48–1.00	0.91	(0.09)	
Neighbourhood watch	Derived from the number of Neighbourhood Watch supporters within each community, scaled per 10,000 population	0.00–0.39	0.10	(0.06)	
Equality					
House price gap	Derived from the Inter Quartile Range of the house price for all houses sold in the Locale in the previous 3 years	0.02–0.96	0.71	(0.19)	
#BeeWell—local environment variables (from 0 to 4)	Derived from the level of agreement reported by participants in the #BeeWell survey (Strongly disagree = 0; Disagree = 1; Neither agree nor disagree = 2; Agree = 3; Strongly Agree = 4)				
I feel safe in the area where I live (8.63% missing)		0–4	2.00	(0.99)	
People around here support each other with their wellbeing (9.31% missing)		0–4	1.62	(1.06)	
You can trust people around here (9.30% missing)		0–4	1.55	(1.11)	
I could ask for help or a favour from neighbours (9.31% missing)		0–4	1.64	(1.21)	
There are good places to spend your free time (e.g. leisure centres, parks, shops) (9.12% missing)		0–4	1.85	(1.10)	
Socio-demographic characteristics of the sample		*n*		%	
School year					
Year 8 (age 12/13)		19,333		53.85	
Year 10 (age 14/15)		16,569		46.15	
Ethnicity					
White		23,101		64.34	
Asian		6558		18.27	
Black		1940		5.40	
Other		3862		10.76	
Missing		441		1.23	
Gender					
Male		14,521		40.45	
Female		14,041		39.11	
Gender diverse		2570		7.16	
Prefer not to say		1912		5.33	
Missing		1858		7.96	
Sex at birth					
Male		18,190		50.67	
Female		17,711		49.43	
Sexual orientation					
Heterosexual		24,171		67.32	
Minority sexual orientation		5061		14.10	
Prefer not to say		3272		9.11	
Missing		3398		9.46	
Free school meals (FSM) eligibility					
No		26,193		72.96	
Yes		9156		25.50	
Missing		553		1.54	
Wellbeing outcome variables					
Life satisfaction (from 0 to 10; 9.69% missing data)		0–10	6.62	(2.51)	
Internalising symptoms (from 0 to 20; 12.27% missing data)		0–20	6.73	(4.73)	

For CWI variables, 1 always represents the optimal score for a given indicator (e.g. better access to schools, smaller house price gap) and 0 the worst

### Analytical approach

Our analysis comprised several stages. First, for each of our outcome variables (life satisfaction and internalising symptoms), a null (empty) multi-level (Level 1: individual; Level 2: neighbourhood) model with no fixed predictors was created to provide a “benchmark” [36] deviance value and allow for the calculation of the ICC (Model 1). Second, we fitted a multi-level model including the socio-demographic co-variates measured at the individual level (i.e. age, ethnicity, gender, sexual orientation, and FSM) (Model 2). To estimate neighbourhood-level variance in adolescents’ wellbeing, we report the ICC for both models. To assess whether inequalities in wellbeing between distinct socio-demographic groups vary across neighbourhoods, we then fitted a model that examined neighbourhood random effects. All the socio-demographic variables were retained in the fixed part of the model, and then each of them was added one at a time in the random part of the model (Model 3). This resulted in six random effects models (Models 3.1–3.6). Likelihood ratio (LR) tests were used to assess model fit improvement [40], which was taken as evidence of neighbourhood random effects.

Finally, to assess whether neighbourhood characteristics are associated with individual wellbeing outcomes, we fitted a series of multi-level models including these variables in the fixed part of the model. First, neighbourhood-level factors grouped by domain (e.g. education and learning, health) were introduced as Level 2 explanatory variables in the same multi-level model (Model 4A, unadjusted). Since there are 8 CWI domains (i.e. education and learning, health, etc.) plus the #BeeWell Local Environment domain, this resulted in 9 models (Models 4A.1–4A.9). We then fitted the same model while controlling for Level 1 socio-demographic characteristics (Model 4B, adjusted). This is, again, 9 models, one per domain (Model 4B.1–4B.9). Second, we explored the unique associations between each neighbourhood characteristic and both wellbeing outcomes in a multi-level model before (Model 5A, unadjusted) and after (Model 5B, adjusted) controlling for Level 1 socio-demographic characteristics. Since there are 17 CWI variables and 5 #BeeWell Local Environment variables, this resulted in 22 unadjusted models (Models 5A.1–5B.22) and 22 adjusted models (Models 5B.1–5B.22).

To facilitate the interpretation of the results, all continuous variables were standardised. Multilevel models were fitted using restricted maximum likelihood estimation. To account for missing data (see levels of missing data in the first column of Table 1), multiple imputation was used. We performed 20 imputations of the data set using a multivariate normal regression approach. Analyses were conducted in STATA 15 [41].

## Results

Table 1 presents the characteristics of the sample of neighbourhoods and adolescents. In terms of CWI neighbourhood characteristic scores, several were uniformly optimal (e.g. mean > 0.9, SD < 0.1) such as access to schools, libraries and health services; areas for leisure; and, social spaces. Others were approaching optimal levels and more variable (e.g. mean > 0.7, SD ≥ 0.1), such as museums, art galleries, theatres; public green space; public space; and, housing price gap. A further tranche were suboptimal and variable (e.g. mean < 0.7, SD ≥ 0.1), such as school quality; GP prescription rates for antidepressants; and, public transport. Finally, several were meagre and variable (e.g. mean < 0.3, SD > 0.1), such as unemployment; free school meals; traffic pollution; air quality; and, neighbourhood watch. #BeeWell local environment variables were all in the low-to-mid range, with the highest neighbourhood average scores for feeling safe and good places to spend free time. Table 2 shows the correlations between the different measures of neighbourhood characteristics.

**Table 2:** Correlation table between life satisfaction, internalising symptoms, CWI neighbourhood characteristics, #BeeWell local environment variables

Variables	(1)	(2)	(3)	(4)	(5)	(6)	(7)	(8)	(9)	(10)	(11)	(12)	(13)	(14)	(15)	(16)	(17)	(18)	(19)	(20)	(21)	(22)	(23)	(24)
(1) Life satisfaction	1.000																							
(2) Internalising symptoms	– 0.613	1.000																						
(3) Access to schools	0.007	– 0.012	1.000																					
(4) School quality	0.007	– 0.002	– 0.042	1.000																				
(5) Access to libraries	– 0.004	0.013	0.226	0.009	1.000																			
(6) Access to health services	0.023	– 0.039	0.241	– 0.126	0.076	1.000																		
(7) GP prescription rates for antidepressants	0.029	– 0.037	0.127	0.163	– 0.030	0.181	1.000																	
(8) Free school meals	0.025	0.010	– 0.064	0.336	0.005	– 0.211	0.325	1.000																
(9) Unemployment	0.025	0.010	– 0.165	0.222	0.000	– 0.204	0.243	0.830	1.000															
(10) Areas for leisure	– 0.003	– 0.024	0.254	– 0.046	0.109	0.323	0.223	– 0.404	– 0.437	1.000														
(11) Museums, art, galleries, music halls, theatres	– 0.006	– 0.007	0.332	– 0.005	0.476	0.055	0.098	– 0.177	– 0.204	0.293	1.000													
(12) Public transport	– 0.005	0.003	0.402	– 0.008	0.271	0.189	0.021	– 0.216	– 0.196	0.285	0.254	1.000												
(13) Public green space	– 0.001	0.013	0.264	0.003	0.178	0.122	– 0.046	– 0.077	– 0.101	0.169	0.184	0.080	1.000											
(14) Public space	– 0.009	0.005	0.234	0.133	0.190	– 0.061	0.065	– 0.069	– 0.133	0.177	0.528	0.117	0.284	1.000										
(15) Air quality	0.004	0.018	– 0.242	0.094	– 0.301	– 0.210	0.066	0.342	0.398	– 0.273	– 0.468	– 0.199	– 0.359	– 0.584	1.000									
(16) Traffic pollution	– 0.016	0.022	– 0.451	0.094	– 0.161	– 0.210	– 0.102	0.056	0.048	– 0.095	– 0.094	– 0.691	– 0.127	– 0.074	0.321	1.000								
(17) Social spaces	– 0.007	0.010	0.287	0.083	0.280	– 0.018	0.035	– 0.074	– 0.164	0.066	0.469	0.156	0.312	0.835	– 0.591	– 0.181	1.000							
(18) Neighbourhood watch	0.006	0.012	– 0.093	0.116	– 0.032	– 0.074	– 0.001	0.311	0.365	0.025	– 0.164	– 0.030	– 0.003	– 0.042	0.288	0.092	– 0.162	1.000						
(19) House price gap	– 0.022	– 0.008	0.035	– 0.335	– 0.146	0.044	– 0.368	– 0.633	– 0.605	0.128	0.008	0.131	– 0.152	– 0.165	– 0.080	– 0.042	– 0.151	– 0.274	1.000					
(20) I feel safe in the area where I live	– 0.045	0.031	0.065	– 0.199	0.076	0.075	– 0.352	– 0.552	– 0.585	0.150	0.128	0.163	0.104	0.146	– 0.378	– 0.066	0.215	– 0.227	0.482	1.000				
(21) People around here support each other with their wellbeing	– 0.055	0.050	0.061	– 0.139	0.105	– 0.016	– 0.362	– 0.394	– 0.449	0.130	0.188	0.196	0.089	0.091	– 0.284	– 0.016	0.122	– 0.154	0.376	0.756	1.000			
(22) You can trust people around here	– 0.054	0.034	0.079	– 0.217	0.154	0.046	– 0.321	– 0.537	– 0.549	0.161	0.196	0.210	0.100	0.167	– 0.389	– 0.046	0.191	– 0.295	0.491	0.832	0.824	1.000		
(23) I could ask for help or a favour from neighbours	– 0.050	0.054	0.021	– 0.108	0.128	– 0.065	– 0.327	– 0.168	– 0.198	– 0.093	0.073	0.045	0.131	0.022	– 0.164	0.067	0.084	– 0.062	0.199	0.517	0.635	0.574	1.000	
(24) There are good places to spend your free time	– 0.041	0.033	– 0.100	– 0.106	– 0.104	– 0.063	– 0.291	– 0.362	– 0.349	0.128	– 0.044	0.109	– 0.160	– 0.237	0.084	0.108	– 0.259	– 0.030	0.484	0.516	0.571	0.528	0.403	1.000

Table 1 also shows that the sample of young people included similar proportions to national figures in terms of the two age groups (54% aged 12/13; 46% aged 14/15), and those reporting their gender as male (40%) or female (39%). 7% of the sample were gender diverse (e.g. non-binary or transgender). Most participants (67%) defined themselves as heterosexual, while 14% reported a minority sexual orientation (e.g. lesbian, gay, bisexual or pansexual). White participants represented 64% of the sample, Asians 18%, Blacks 5%, with those of other ethnic groups making up 11%. Approximately 25% were eligible for FSM in the last 6 years.

Table 3 shows that the proportion of the total variance in young people’s wellbeing attributable to differences between neighbourhoods is small but statistically significant (Models 1 and 2). The ICC is approximately twice the size for internalising symptoms (1.17%) as it is for life satisfaction (0.61%). After including the socio-demographic co-variates in the model, the ICCs reduced approximately by half for both outcomes.

**Table 3:** Intraclass correlation coefficients for neighbourhood-level variance in adolescents’ wellbeing (35,902 adolescents, 243 neighbourhoods)

	Unadjusted models			Adjusted model for year group, ethnicity, gender, sexuality and FSM		
	(Model 1)			(Model 2)		
	ICC	(95% CI)	*p*	ICC	(95% CI)	*p*
Life satisfaction	0.0061	(0.0039–0.0096)	< .0001	0.0034	(0.0018–0.0066)	< 0.0001
Internalising symptoms	0.0117	(0.0084–0.0162)	< .0001	0.0058	(0.0036–0.0094)	< 0.0001

LR tests assessing model fit improvement when adding random effects in Model 3 (see Models 3.1–3.6 in Table A1 in Supplementary Appendix 1) provide evidence that neighbourhoods shape wellbeing inequalities. Specifically, we find evidence that sex, gender, and sexuality inequalities in life satisfaction and internalising symptoms vary across neighbourhoods (*p* < 0.05).

Table 4 presents the associations between the various groups of neighbourhood factors and individual wellbeing outcomes (Models 4A and 4B). In these and other tables, regression coefficients are standardised (e.g. in Model 4B, a 1 standard deviation (SD) increase in perceived local support for wellbeing is associated with a 0.231 SD decrease in internalising symptoms). For CWI variables, no significant associations were found in the areas of education and learning; economy, work and employment; and transport, mobility and connectivity (all *p* > 0.05). In the health domain, better access to health services and lower GP prescription rates for antidepressants in neighbourhoods were associated with higher life satisfaction (respectively, regression coefficients *B* = 0.015; *B* = 0.024) and lower internalising symptoms (*B* = − 0.025; *B* = − 0.029) at the individual level. In the culture, heritage and leisure domain, better access to areas for leisure in neighbourhoods were associated with lower internalising symptoms at the individual level (*B* = − 0.022). However, these associations became non-significant after controlling for socio-demographic characteristics. In the housing, space and environment domain, better air quality was associated with higher internalising symptoms (*B* = 0.025), but this counter-intuitive association became non-significant after controlling for socio-demographic characteristics. In the domain of relationships and trust, another counter-intuitive result emerged, as better access to social spaces were associated with greater internalising symptoms at the individual level, though only when controlling for socio-demographic characteristics (*B* = 0.014). Finally, in the equality domain, smaller neighbourhood house price gap was associated with lower life satisfaction at the individual level (*B* = − 0.028); this counter-intuitive association remained statistically significant even after controlling for socio-demographic characteristics (*B* = − 0.017).

**Table 4:** Results from multi-level models with random intercepts showing grouped associations between different types of neighbourhood factors and adolescents’ wellbeing

	Life satisfaction						Internalising symptoms					
	Unadjusted			Adjusted models for year group, ethnicity, gender, sexuality and FSM			Unadjusted			Adjusted models for year group, ethnicity, gender, sexuality and FSM		
	(Model 4A)			(Model 4B)			(Model 4A)			(Model 4B)		
	Coefficient	(95% CI)	*p*	Coefficient	(95% CI)	*p*	Coefficient	(95% CI)	*p*	Coefficient	(95% CI)	*p*
CWI—community variables												
Education and learning												
Access to schools	0.003	(– 0.011 to 0.017)	0.694	0.003	(– 0.010 to 0.015)	0.646	– 0.004	(– 0.020 to 0.011)	0.594	0.001	(– 0.013 to 0.013)	0.987
School quality	0.010	(– 0.005 to 0.026)	0.199	0.006	(– 0.007 to 0.020)	0.359	– 0.003	(– 0.021 to 0.016)	0.776	– 0.005	(– 0.019 to 0.008)	0.446
Access to libraries	– 0.001	(– 0.017 to 0.015)	0.907	0.000	(– 0.013 to 0.014)	0.958	0.007	(– 0.011 to 0.025)	0.428	0.003	(– 0.105 to 0.164)	0.664
Health												
Access to health services	**0.015**	(0.001 to 0.030)	0.039	0.009	(– 0.005 to 0.022)	0.199	**– 0.025**	(– 0.041 to – 0.008)	0.003	– 0.009	(– 0.019 to 0.005)	0.206
GP prescription rates for antidepressants	**0.024**	(0.009 to 0.039)	0.002	0.013	(– 0.001 to 0.026)	0.063	**– 0.029**	(– 0.046 to – 0.011)	0.001	– 0.009	(– 0.022 to 0.005)	0.222
Economy, work and employment												
Unemployment	0.011	(– 0.013 to 0.036)	0.371	0.006	(– 0.016 to 0.028)	0.581	– 0.007	(– 0.036 to 0.020)	0.598	– 0.009	(– 0.031 to 0.013)	0.418
Free school meals	0.016	(– 0.009 to 0.042)	0.272	0.010	(– 0.013 to 0.032)	0.401	0.007	(– 0.023 to 0.036)	0.648	0.003	(– 0.019 to 0.026)	0.771
Culture, heritage and leisure												
Areas for leisure	– 0.004	(– 0.017 to 0.016)	0.955	– 0.004	(– 0.018 to 0.010)	0.601	**– 0.022**	(– 0.041 to – 0.003)	0.021	– 0.005	(– 0.020 to 0.009)	0.455
Museums, art galleries, music halls, theatres	– 0.004	(– 0.021 to 0.011)	0.593	– 0.002	(– 0.016 to 0.012)	0.759	0.001	(– 0.017 to 0.019)	0.928	0.006	(– 0.007 to 0.020)	0.354
Transport, mobility and connectivity												
Public transport	– 0.006	(– 0.021 to 0.008)	0.405	– 0.004	(– 0.016 to 0.009)	0.587	0.005	(– 0.012 to 0.021)	0.574	0.011	(– 0.001 to 0.024)	0.072
Housing, space and environment												
Public green space	0.004	(– 0.012 to 0.019)	0.653	0.003	(– 0.011 to 0.016)	0.707	0.013	(– 0.004 to 0.030)	0.121	0.010	(– 0.004 to 0.023)	0.156
Public space	– 0.010	(– 0.029 to 0.008)	0.271	– 0.005	(– 0.022 to 0.011)	0.512	0.016	(– 0.005 to 0.037)	0.128	0.013	(– 0.003 to 0.029)	0.117
Traffic pollution	– 0.014	(– 0.029 to 0.001)	0.074	– 0.011	(– 0.024 to 0.003)	0.111	0.009	(– 0.008 to 0.026)	0.298	0.004	(– 0.009 to 0.017)	0.544
Air quality	0.002	(– 0.018 to 0.021)	0.870	0.001	(– 0.016 to 0.019)	0.877	**0.025**	(– 0.003 to 0.047)	0.028	0.007	(– 0.010 to 0.025)	0.339
Relationships and trust												
Social spaces	– 0.006	(– 0.021 to 0.009)	0.406	– 0.001	(– 0.014 to 0.012)	0.897	0.013	(– 0.004 to 0.030)	0.141	**0.014**	(0.001 to 0.027)	0.031
Neighbourhood watch	0.008	(– 0.007 to 0.023)	0.318	0.005	(– 0.008 to 0.019)	0.435	0.001	(– 0.016 to 0.019)	0.882	– 0.005	(– 0.018 to 0.008)	0.460
Equality												
House price gap	**– 0.028**	(– 0.043 to – 0.012)	0.001	**– 0.017**	(– 0.031 to – 0.004)	0.012	0.000	(– 0.018 to 0.018)	0.999	0.000	(– 0.013 to 0.014)	0.959
#BeeWell—local environment variables												
I feel safe in the area where I live	– 0.072	(– 0.201 to 0.058)	0.277	– 0.113	(– 0.232 to 0.006)	0.062	0.005	(– 0.152 to 0.161)	0.953	0.004	(– 0.081 to 0.167)	0.500
People around here support each other with their wellbeing	0.155	(– 0.016 to 0.326)	0.075	**0.171**	(– 0.014 to 0.328)	0.033	**– 0.296**	(– 0.499 to 0.094)	0.004	**– 0.231**	(– 0.391 to 0.072)	0.004
You can trust people around here	**0.163**	(– 0.005 to 0.320)	0.043	0.144	(– 0.002 to 0.291)	0.053	0.149	(– 0.041 to 0.339)	0.123	0.004	(– 0.149 to 0.158)	0.959
I could ask for help or a favour from neighbours	**0.142**	(0.023 to 0.261)	0.019	0.069	(– 0.041 to 0.179)	0.221	**– 0.226**	(– 0.372 to – 0.081)	0.002	– 0.083	(– 0.200 to 0.033)	0.160
There are good places to spend your free time	0.078	(– 0.016 to 0.172)	0.104	0.054	(– 0.033 to 0.141)	0.221	– 0.076	(– 0.190 to 0.037)	0.189	– 0.032	(– 0.120 to 0.056)	0.475

Statistically significant effects (*p* < 0.05) are highlighted in bold

In terms of #BeeWell local environment items, higher levels of perceived helpfulness of neighbours were associated with higher life satisfaction (*B* = 0.142) and lower internalising symptoms at the individual level (*B* = − 0.226), but only in the unadjusted model. Higher levels of trust in local people were associated with higher life satisfaction (*B* = 0.142), but only in the unadjusted model. Higher levels of neighbourhood wellbeing support from local people were associated with higher life satisfaction, but only after controlling for socio-demographic characteristics (*B* = 0.163), and lower internalising symptoms before (*B* = − 0.296), and after controlling for socio-demographic characteristics (*B* = − 0.231).

Our analysis of unique associations in Table 5 (Models 5A and 5B) reveals some additional relationships between neighbourhood characteristics and adolescents’ wellbeing outcomes that may not be observed in Models 4A and 4B (Table 4) due to collinearity issues. In the CWI domain of economy, work, and employment, lower neighbourhood unemployment (*B* = 0.024) and FSM eligibility rates (*B* = 0.026) were associated with higher life satisfaction at the individual level, even after controlling for socio-demographic characteristics (*B* = 0.014; *B* = 0.014). In the #BeeWell local environment items, higher neighbourhood levels of feeling safe (*B* = 0.248), perceived support for wellbeing among local people *B* = 0.383), trust in local people (*B* = 0.314), neighbour helpfulness (*B* = 0.352), and good places to spend free time (*B* = 0.256), each were associated with higher life satisfaction and lower internalising symptoms (feeling safe *B* = − 0.191; perceived support for wellbeing *B* = − 0.330; trust in local people *B* = − 0.201; neighbour helpfulness *B* = − 0.352; good places to spend free time *B* = − 0.225) at the individual level.

**Table 5:** Unique associations from multi-level models with random intercepts between neighbourhood factors and adolescents’ wellbeing

	Unadjusted			Adjusted models for year group, ethnicity, gender, sexuality and FSM			Unadjusted			Adjusted models for year group, ethnicity, gender, sexuality and FSM		
	(Model 5A)			(Model 5B)			(Model 5A)			(Model 5B)		
	Coefficient	(95% CI)	*p*	Coefficient	(95% CI)	*p*	Coefficient	(95% CI)	*p*	Coefficient	(95% CI)	*p*
CWI—community variables												
Education and learning												
Access to schools	0.002	(− 0.011 to 0.016)	0.725	0.003	(− 0.009 to 0.015)	0.642	− 0.003	(− 0.018 to 0.012)	0.706	0.001	(− 0.012 to 0.013)	0.927
School quality	0.010	(− 0.005 to 0.026)	0.203	0.006	(− 0.007 to 0.020)	0.363	− 0.002	(− 0.020 to 0.016)	0.809	− 0.005	(− 0.019 to 0.008)	0.452
Access to libraries	0.000	(− 0.015 to 0.015)	0.955	0.001	(− 0.012 to 0.014)	0.859	0.006	(− 0.011 to 0.023)	0.494	0.003	(− 0.010 to 0.016)	0.673
Health												
Access to health services	**0.018**	(0.003 to 0.033)	0.007	0.010	(− 0.002 to 0.023)	0.130	**− 0.028**	(− 0.044 to − 0.011)	0.001	− 0.010	(− 0.023 to 0.004)	0.156
GP prescription rates for antidepressants	**0.026**	(0.011 to 0.041)	0.001	**0.014**	(0.000 to 0.027)	0.042	**− 0.032**	(− 0.050 to − 0.014)	0.000	− 0.010	(− 0.023 to 0.004)	0.167
Economy, work and employment												
Unemployment	**0.024**	(0.009 to 0.038)	0.001	**0.014**	(0.001 to 0.027)	0.038	− 0.002	(− 0.019 to 0.014)	0.787	− 0.007	(− 0.020 to 0.007)	0.329
Free school meals	**0.026**	(0.011 to 0.041)	0.001	**0.014**	(0.001 to 0.028)	0.031	0.001	(− 0.017 to 0.018)	0.948	− 0.004	(− 0.018 to 0.009)	0.544
Culture, heritage and leisure												
Areas for leisure	− 0.002	(− 0.018 to 0.014)	0.822	− 0.004	(− 0.018 to 0.009)	0.526	**− 0.022**	(− 0.040 to − 0.004)	0.017	− 0.004	(− 0.017 to 0.010)	0.608
Museums, art galleries, music halls, theatres	− 0.005	(− 0.020 to 0.011)	0.562	− 0.003	(− 0.017 to 0.010)	0.636	− 0.005	(− 0.023 to 0.012)	0.543	0.005	(− 0.008 to 0.018)	0.458
Transport, mobility and connectivity												
Public transport	− 0.006	(− 0.021 to 0.008)	0.405	− 0.004	(− 0.016 to 0.009)	0.587	0.005	(− 0.012 to 0.021)	0.574	0.011	(− 0.001 to 0.024)	0.072
Housing, space and environment												
Public green space	− 0.008	(− 0.024 to 0.006)	0.257	− 0.004	(− 0.018 to 0.009)	0.519	0.009	(− 0.007 to 0.025)	0.269	0.011	(− 0.002 to 0.024)	0.097
Public space	0.002	(− 0.013 to 0.016)	0.796	0.002	(− 0.011 to 0.015)	0.754	0.005	(− 0.013 to 0.0222)	0.585	0.010	(− 0.002 to 0.023)	0.107
Traffic pollution	− 0.013	(− 0.027 to 0.002)	0.085	− 0.010	(− 0.023 to 0.002)	0.110	0.013	(− 0.004 to 0.029)	0.127	0.004	(− 0.008 to 0.017)	0.554
Air quality	0.002	(− 0.013 to 0.017)	0.802	0.000	(− 0.013 to 0.013)	0.955	0.014	(− 0.004 to 0.031)	0.119	− 0.002	(− 0.015 to 0.011)	0.756
Relationships and trust												
Social spaces	− 0.007	(− 0.022 to 0.007)	0.318	− 0.002	(− 0.015 to 0.011)	0.812	0.013	(− 0.004 to 0.029)	0.142	**0.015**	(0.002 to 0.028)	0.022
Neighbourhood Watch	0.008	(− 0.006 to 0.024)	0.255	0.005	(− 0.008 to 0.018)	0.419	− 0.001	(− 0.018 to 0.017)	0.823	− 0.007	(− 0.020 to 0.006)	0.296
Equality												
House price gap	**− 0.028**	(− 0.043 to − 0.012)	0.001	**− 0.017**	(− 0.031 to − 0.004)	0.012	0.000	(− 0.018 to 0.018)	0.999	0.000	(− 0.013 to 0.014)	0.959
#BeeWell—local environment variables												
I feel safe in the area where I live	**0.248**	(0.172 to 0.324)	0.000	**0.160**	(0.090 to 0.231)	0.000	**− 0.191**	(− 0.282 to − 0.100)	0.000	**− 0.148**	(− 0.220 to − 0.077)	0.000
People around here support each other with their wellbeing	**0.383**	(0.294 to 0.471)	0.000	**0.282**	(0.202 to 0.362)	0.000	**− 0.330**	(− 0.436 to − 0.224)	0.000	**− 0.259**	(− 0.341 to − 0.177)	0.000
You can trust people around here	**0.314**	(0.238 to 0.391)	0.000	**0.229**	(0.158 to 0.299)	0.000	**− 0.201**	(− 0.294 to − 0.108)	0.000	**− 0.179**	(− 0.252 to − 0.107)	0.000
I could ask for help or a favour from neighbours	**0.352**	(0.257 to 0.448)	0.000	**0.234**	(0.147 to 0.322)	0.000	**− 0.352**	(− 0.466 to − 0.238)	0.000	**− 0.222**	(− 0.313 to − 0.130)	0.000
There are good places to spend your free time	**0.256**	(0.172 to 0.339)	0.000	**0.177**	(0.102 to 0.252)	0.000	**− 0.225**	(− 0.321 to − 0.129)	0.000	**− 0.152**	(− 0.226 to − 0.078)	0.000

Statistically significant effects (*p*<0.05) are highlighted in bold

The above pattern held even after controlling for socio-demographic characteristics, for both life satisfaction (feeling safe *B* = 0.160; perceived support for wellbeing *B* = 0.282; trust in local people *B* = 0.229; neighbour helpfulness *B* = 0.234; good places to spend free time *B* = 0.177) and for internalising symptoms (feeling safe *B* = − 0.148; perceived support for wellbeing *B* = − 0.259; trust in local people *B* = − 0.179; neighbour helpfulness *B* = − 0.222; good places to spend free time *B* = − 0.152).

## Discussion

Considering the scarcity of research investigating neighbourhood effects on adolescent wellbeing (compared to other developmental contexts such as family and school), we studied the extent to which adolescents’ life satisfaction and internalising symptoms vary across neighbourhoods in Greater Manchester (England), and which neighbourhood characteristics are associated with these wellbeing outcomes at the individual level. Data were analysed from 35,902 12–15-year-olds across 243 neighbourhoods, whose characteristics spanned a range of community-level factors in multiple domains.

While the relative lack of research on neighbourhood effects on wellbeing means we must be cautious in our inferences, our findings were in line with the limited previous research that found small but statistically significant neighbourhood effects [12–14, 24]. However, with 1.17% and 0.61% of the variation in internalising symptoms and life satisfaction (respectively) explained at the neighbourhood level, our estimates are at the lower end of the 1–5% range typically observed in these studies, and substantially lower than neighbourhood wellbeing ICCs observed for adults, which can reach 7% or greater [15–17]. One potential explanation relates to the timing of the study, coming as it did after a sustained period of societal restrictions due to the Covid-19 pandemic, which will likely have disrupted some the processes and mechanisms through which neighbourhoods influence adolescent wellbeing. Alternatively, the boundaries of our neighbourhood units themselves (‘seamless locales’) may have played a role. Though these were designed to reflect how people familiar with a given area would divide them [34], this involved input from adults rather than adolescents. A similar exercise undertaken with young people may well have led to different neighbourhood boundaries and, thus, different ICCs [28]. This may also explain why neighbourhood wellbeing effects appear to be larger among adults. Even though they are typically based on administrative units, these are likely more aligned to adult perceptions of geographic local area boundaries than those of adolescents. More broadly, this issue speaks to the ‘uncertain geographic context’ problem noted earlier [29]. Accordingly, in a future study, we intend to consider different available geographic units to examine the extent to which these influence the presence and magnitude of neighbourhood effects on adolescent wellbeing. Given the aforementioned note regarding the impact of the pandemic, we will also examine whether this changes over time.

That the ICC for internalising symptoms was approximately double that of life satisfaction is consistent with the extant evidence base [12–14]. By way of explanation, Snedker and Herting [24] speculate that the neighbourhood context is particularly pertinent to adolescent distress. Using a stress process lens, they argue that, “certain ecological conditions, such as neighbourhood economic disadvantage and residential instability, influence the type and level of stress exposure and available resources for coping” (p.697). This explanation finds mixed support in our analysis; while we found that access to health services (arguably a good proxy for coping resource) were associated with lower internalising symptoms in our unadjusted models, none of our neighbourhood markers of socio-economic disadvantage (e.g. unemployment, free school meals) did so, even in the unique association models. We also note that our neighbourhood markers of socio-economic disadvantage actually were associated with life satisfaction in our unique association models, and—in view of Table 2—collinearity may be the reason why associations were not observed in the grouped associations model. This finding aligns with a recent systematic review, which reported stronger associations between neighbourhood socio-economic disadvantage and wellbeing indicators than for internalising symptoms [20]. It is possible to reconcile these apparently contradictory positions by considering that neighbourhood differences may explain more *variance* in symptoms than in wellbeing indicators, but that the specific neighbourhood *measures* of socio-economic deprivation in a given study may not always have sufficient explanatory power. As with other indicators, this may be because existing measures of socio-economic deprivation are essentially too ‘blunt’, failing to adequately capture young people’s active roles in forming views on their material needs and assessing their comparative socio-economic status [42, 43].

We also found evidence of wellbeing inequalities varying across neighbourhoods (sex, gender, and sexuality inequalities in both life satisfaction and internalising symptoms). A recent UK study, albeit one involving substantially bigger clusters (local areas) and older adolescents (aged 16–24), found similar evidence for inequalities in loneliness with regard to sex and sexual orientation [44]. Evidence that wellbeing disadvantage among minority groups may be affected by where young people live provokes a call for more nuanced responses that identify the neighbourhoods with increased vulnerability and inequality. It also suggests that targeted, hyper-local responses in these areas may be as important, if not more so, than national initiatives, since the former are better placed to examine and respond to the complex contextual factors that underpin (and reinforce) exacerbated wellbeing inequalities [45].

Several neighbourhood characteristics were associated with wellbeing outcomes, but differences across model specification were observed (e.g. adjusted vs unadjusted; unique associations vs grouped domains). However, higher levels of perceived wellbeing support from local people were associated with lower internalising symptoms in all models, and this is unlikely to be an artefact of same source bias given the aggregation to the neighbourhood level [30]. This is aligned with previous studies noting the importance of neighbourhood social capital to adolescent wellbeing [13, 25, 26] This robust finding connects to work on neighbourhood social-interactive processes [46] that are theorised to impact wellbeing, and more specifically the concept of ‘neighbourliness' [47], defined as when local residents, “engage in warm, caring, and mutually supportive relationships… one can think of neighbourliness as the behavioural expression of neighbourhood social capital” (p.311). Place-based responses that emphasise and promote a sense of belonging to the local community, social cohesion, integration and inclusivity, and opportunities and structures for social support, may, therefore, yield benefits in terms of reduced internalising symptoms [48].

Furthermore, neighbourhoods with better access to health services and lower GP antidepressant prescription rates were consistently associated with higher life satisfaction and lower internalising symptoms at the individual level in our unadjusted models in both the grouped domains and unique associations analyses. Good access to health services may be thought of as a local institutional resource [46] that benefits population health. Our findings are particularly timely and salient given evidence that the Covid-19 pandemic has exacerbated access problems in general practice [49], combined with data indicating that, even before the pandemic, one quarter of referrals to child and adolescent mental health services were rejected, and average wait times were double the government’s proposed four-week standard [50]. Clearly then, work which seeks to address the social determinants of wellbeing inequalities should be a priority area for action, as should increased investment and resourcing in the public health system [45]. We give this issue particular emphasis given the parallel finding pertaining to GP antidepressant prescription rates. The established links between parental mental health and child wellbeing [51] notwithstanding, when viewed as a proxy for levels of mental health need in a neighbourhood, the notion of ‘double disadvantage’ is brought to mind, i.e. a potential compound effect of high levels of need *and* poor access to health services in certain neighbourhoods.

Our analyses also provided evidence that what is available to adolescents in their free time is associated with their wellbeing (e.g. CWI areas for leisure and internalising symptoms; perceptions of having good places to spend free time and both internalising symptoms and life satisfaction), a finding that has support in other recent research [52]. As with access to health services, areas for leisure and free time can be thought of as local institutional resources [46] that promote population health. In this instance, these particular resources serve a number of important functions pertaining to adolescent development, including supporting the process of identity formation, promoting autonomy and the development of decision-making skills, socialisation, and playing a buffering role against the effects of stress [53]. Increased investment in free/leisure time infrastructure for young people is therefore warranted, but insufficient in and of itself. Access to and participation in leisure time activities varies by age, sex, family structure and socio-economic status, and any community-based initiatives would need to take account of these disparities, in addition to ensuring that an appropriately wide range of leisure time resources are made available [52].

In considering the above findings and their implications, it is important to note several of the associations between neighbourhood characteristics and individual outcomes did not hold in our adjusted models, in which we accounted for a range of individual socio-demographic characteristics (e.g. FSM, ethnicity). One interpretation is that these findings may be driven more strongly by these individual characteristics than the neighbourhood-level variables that were our primary explanatory variables. However, adjusting for individual differences in socio-demographic characteristics in multi-level modelling implies controlling for differences across clusters (neighbourhoods) on these individual-level variables, indicating that the socio-demographic composition of the neighbourhood plays an important role in explaining the fixed effects. At the same time, the fact that some associations were observed in the unique associations models (Table 5) but not in the grouped associations models (Table 4) may be due to collinearity. In view of Table 2, this would affect the socio-economic variables (unemployment and FSM) and the #BeeWell Local Environment variables relating to social capital (perceived social support and trust). Overall, given the differences observed across model specifications (unique associations VS grouped associations; adjusted VS unadjusted models), we suggest that the primary focus in terms of implications should be on those that are more consistently associated with adolescent wellbeing across models, this is social capital measures of perceived social support and trust in local people.

The current study has a number of strengths, including a very large sample whose composition closely mirrored the local population from which it was drawn; wide range of neighbourhood measures spanning multiple domains and making use of both participant-assessed and administrative neighbourhood data; and application of differing model specifications to test the sensitivity of neighbourhood effects to modelling decisions. However, there are also several limitations that should be borne in mind. First, our study focused specifically on the 12–15 age range and future research should explore the influences of neighbourhoods on wellbeing of older (i.e. 16+ year olds) and younger (i.e. < 12 year olds) adolescents. Second, the study was cross-sectional in nature, limiting causal inference. Future research can address this issue using a longitudinal design, potentially involving path analyses to provide insights into the mechanisms that underpin neighbourhood effects (e.g. socio-economic disadvantage influencing internalising symptoms via lower cumulative social support and perceptions of social cohesion [48]). Third, as noted above, Table 2 shows strong correlations between some #BeeWell Local Environment variables as well as between the socio-economic variables (unemployment and FSM), which calls for caution when interpreting the results of the group associations models (Model 4 in Table 4) for these specific variables. Finally, CWI variables were not necessarily attuned to the needs of adolescents (e.g. social spaces considered the distance to the nearest pub, café, community centre, and playground). This may explain the rather limited number of noteworthy associations (or, in the specific cases of social spaces, house price gap and air quality, the counter-intuitive associations that were observed). Future research in which young people are consulted about the characteristics of their local areas that are most meaningful to them in terms of their wellbeing, leading to the co-production of adolescent-derived neighbourhood indices, is, therefore, warranted. Indeed, this could be undertaken in tandem with the aforementioned work to better understand how young people conceptualise the geographic boundaries of their neighbourhoods.

In conclusion, the current study demonstrates that neighbourhoods account for a small but significant proportion of the variance in adolescent life satisfaction and internalising symptoms. Our analysis of fixed effects reveals some noteworthy associations between adolescent wellbeing and particular neighbourhood characteristics, especially those pertaining to social capital. Evidence on the relevance of neighbourhoods to wellbeing inequalities across socio-demographic groups, and that some of their characteristics are associated with these outcomes at the individual level, have important implications for intervention. However, future research which adopts a longitudinal design and uses adolescent-derived indices of neighbourhoods and their characteristics is needed to more fully understand the role of place as a determinant of young people’s wellbeing.

### Supplementary Information

Below is the link to the electronic supplementary material.

Supplementary file1 (DOCX 37 KB)

## Data Availability

An anonymised version of the #BeeWell survey responses will be made publicly available in 2026. Due to ethical constraints this cannot be brought forward since participants have been given the right to withdraw their data until this point necessitating the need to maintain a securely stored pseudonymised version until this point. In addition, the non-self report data such as sex and free school meal eligibility will never be shared publicly due to the agreement in place with the local authorities who provided it.
