# Supplementary material for: This is the place: a multi-level analysis of neighbourhood correlates of adolescent wellbeing
Source: Soc Psychiatry Psychiatr Epidemiol. 2023 Aug 22;59(6):929–46. doi: 10.1007/s00127-023-02531-y (PMC11116214; doi:10.1007/s00127-023-02531-y)
Supplement: Supplementary file 1 — Supplementary file1 (DOCX 37 KB) [file 127_2023_2531_MOESM1_ESM.docx]

**Appendix 1**

| Table A1. Results from Multilevel Models with Random Slopes Showing Neighbourhood Random Effects in Adolescents' Wellbeing for Socio-Demographic Variables (35,902 adolescents, 243 neighbourhoods) | | | | | | | | | | | | | | | | | |
| --- | --- | --- | --- | --- | --- | --- | --- | --- | --- | --- | --- | --- | --- | --- | --- | --- | --- |
|  | Life satisfaction | | | | | | | | | | | | | | | | |
|  | Year group random effect | |  | Ethnicity random effect | |  | Sex random effect | |  | Gender random effect | |  | Sexuality random effect | |  | FSM random effect | |
|  | (Model 3.1) | |  | (Model 3.2) | |  | (Model 3.3) | |  | (Model 3.4) | |  | (Model 3.5) | |  | (Model 3.6) | |
|  | b | S.E. |  | b | S.E. |  | b | S.E. |  | b | S.E. |  | b | S.E. |  | b | S.E. |
| *Fixed:* |  |  |  |  |  |  |  |  |  |  |  |  |  |  |  |  |  |
| Year 10 (ref.: year 8) | -0.259*** | (0.011) |  | -0.259*** | (0.011) |  | -0.256*** | (0.011) |  | -0.259*** | (0.011) |  | -0.259*** | (0.011) |  | -0.259*** | (0.011) |
| Ethnicity - Black (ref.: white) | 0.023 | (0.026) |  | 0.022 | (0.026) |  | 0.024 | (0.026) |  | 0.021 | (0.026) |  | 0.022 | (0.026) |  | 0.022 | (0.026) |
| Ethnicity - Asian (ref.: white) | 0.053** | (0.016) |  | 0.050** | (0.016) |  | 0.048** | (0.016) |  | 0.047** | (0.016) |  | 0.049** | (0.016) |  | 0.053** | (0.016) |
| Ethnicity - Other (ref.: white) | -0.051** | (0.019) |  | -0.053** | (0.019) |  | -0.048* | (0.018) |  | -0.053** | (0.018) |  | -0.052** | (0.018) |  | -0.051** | (0.019) |
| Sex at birth - Female (ref.: male) |  |  |  |  |  |  | -0.368*** | (0.013) |  |  |  |  |  |  |  |  |  |
| Gender - Female (ref.: male) | -0.366*** | (0.012) |  | -0.366*** | (0.012) |  |  |  |  | -0.367*** | (0.013) |  | -0.366*** | (0.012) |  | -0.366*** | (0.012) |
| Gender - Gender diverse (ref.: male) | -0.506*** | (0.023) |  | -0.506*** | (0.023) |  |  |  |  | -0.510*** | (0.025) |  | -0.505*** | (0.023) |  | -0.506*** | (0.023) |
| Gender - Prefer not to say (ref.: male) | -0.265*** | (0.026) |  | -0.265*** | (0.026) |  |  |  |  | -0.269*** | (0.029) |  | -0.266*** | (0.026) |  | -0.265*** | (0.026) |
| Sexuality - Minority sexual orientation (ref.: heterosexual) | -0.550*** | (0.017) |  | -0.550*** | (0.017) |  | -0.617*** | (0.015) |  | -0.548*** | (0.017) |  | -0.553*** | (0.017) |  | -0.550*** | (0.017) |
| Sexuality - Prefer not to say  (ref.: heterosexual) | -0.170*** | (0.020) |  | -0.170*** | (0.020) |  | -0.198*** | (0.019) |  | -0.171*** | (0.020) |  | -0.179*** | (0.022) |  | -0.170*** | (0.020) |
| FSM (ref.: no FSM) | -0.138*** | (0.013) |  | -0.139*** | (0.013) |  | -0.142*** | (0.013) |  | -0.138*** | (0.013) |  | -0.139*** | (0.013) |  | -0.138*** | (0.013) |
| *Random:* |  |  |  |  |  |  |  |  |  |  |  |  |  |  |  |  |  |
|  | 0.055 | |  | 0.054 | |  | 0.046 | |  | 0.042 | |  | 0.055 | |  | 0.055 | |
| Neighbourhood level (S.D., 95% C.I., S.E.) | (0.040 to 0.077) | |  | (0.038 to 0.077) | |  | (0.029 to 0.073) | |  | (0.024 to 0.073) | |  | (0.036 to 0.075) | |  | (0.039 to 0.077) | |
|  | (0.010) | |  | (0.010) | |  | (0.011) | |  | (0.012) | |  | (0.010) | |  | (0.010) | |
|  | 0.000 | |  |  |  |  |  |  |  |  |  |  |  |  |  |  |  |
| Year group random effect (S.D., 95% C.I., S.E.) | (0.000 to 0.000) | |  |  |  |  |  |  |  |  |  |  |  |  |  |  |  |
|  | (0.000) | |  |  |  |  |  |  |  |  |  |  |  |  |  |  |  |
|  |  |  |  | 0.015 | |  |  |  |  |  |  |  |  |  |  |  |  |
| Ethnicity random effect (S.D., 95% C.I., S.E.) |  |  |  | (0.002 to 0.094) | |  |  |  |  |  |  |  |  |  |  |  |  |
|  |  |  |  | (0.014) | |  |  |  |  |  |  |  |  |  |  |  |  |
|  |  |  |  |  |  |  | 0.076 | |  |  |  |  |  |  |  |  |  |
| Sex random effect (S.D., 95% C.I., S.E.) |  |  |  |  |  |  | (0.053 to 0.108) | |  |  |  |  |  |  |  |  |  |
|  |  |  |  |  |  |  | (0.014) | |  |  |  |  |  |  |  |  |  |
|  |  |  |  |  |  |  |  |  |  | 0.052 | |  |  |  |  |  |  |
| Gender random effect (S.D., 95% C.I., S.E.) |  |  |  |  |  |  |  |  |  | (0.037 to 0.072) | |  |  |  |  |  |  |
|  |  |  |  |  |  |  |  |  |  | (0.009) | |  |  |  |  |  |  |
|  |  |  |  |  |  |  |  |  |  |  |  |  | 0.052 | |  |  |  |
| Sexuality random effect (S.D., 95% C.I., S.E.) |  |  |  |  |  |  |  |  |  |  |  |  | (0.036 to 0.075) | |  |  |  |
|  |  |  |  |  |  |  |  |  |  |  |  |  | (0.013) | |  |  |  |
|  |  |  |  |  |  |  |  |  |  |  |  |  |  |  |  | 0.044 | |
| FSM random effect (S.D., 95% C.I., S.E.) |  |  |  |  |  |  |  |  |  |  |  |  |  |  |  | (0.011 to 0.171) | |
|  |  |  |  |  |  |  |  |  |  |  |  |  |  |  |  | (0.030) | |
|  | 0.940 | |  | 0.940 | |  | 0.940 | |  | 0.940 | |  | 0.940 | |  | 0.940 | |
| Individual level (S.D., 95% C.I., S.E.) | (0.933 to 0.948) | |  | (0.932 to 0.948) | |  | (0.932 to 0.947) | |  | (0.932 to 0.947) | |  | (0.932 to 0.947) | |  | (0.932 to 0.948) | |
|  | (0.04) | |  | (0.004) | |  | (0.004) | |  | (0.004) | |  | (0.004) | |  | (0.004) | |
|  |  |  |  |  |  |  |  |  |  |  |  |  |  |  |  |  |  |
|  | Internalising symptoms | | | | | | | | | | | | | | | | |
|  | Year group random effect | |  | Ethnicity random effect | |  | Sex random effect | |  | Gender random effect | |  | Sexuality random effect | |  | FSM random effect | |
|  | (Model 3) | |  | (Model 3) | |  | (Model 3) | |  | (Model 3) | |  | (Model 3) | |  | (Model 3) | |
|  | b | S.E. |  | b | S.E. |  | b | S.E. |  | b | S.E. |  | b | S.E. |  | b | S.E. |
| *Fixed:* |  |  |  |  |  |  |  |  |  |  |  |  |  |  |  |  |  |
| Year 10 (ref.: year 8) | 0.078*** | (0.012) |  | 0.077*** | (0.011) |  | 0.079*** | (0.011) |  | 0.078*** | (0.011) |  | 0.078*** | (0.011) |  | 0.077*** | (0.011) |
| Ethnicity - Black (ref.: white) | -0.161*** | (0.026) |  | -0.159*** | (0.026) |  | -0.164*** | (0.026) |  | -0.159*** | (0.026) |  | -0.159*** | (0.026) |  | -0.160*** | (0.026) |
| Ethnicity - Asian (ref.: white) | -0.200*** | (0.016) |  | -0.193*** | (0.017) |  | -0.195*** | (0.015) |  | -0.196*** | (0.015) |  | -0.196*** | (0.016) |  | -0.200*** | (0.016) |
| Ethnicity - Other (ref.: white) | -0.072*** | (0.018) |  | -0.067*** | (0.019) |  | -0.078*** | (0.018) |  | -0.070*** | (0.018) |  | -0.071*** | (0.018) |  | -0.071*** | (0.018) |
| Sex at birth - Female (ref.: male) |  |  |  |  |  |  | 0.588*** | (0.012) |  |  |  |  |  |  |  |  |  |
| Gender - Female (ref.: male) | 0.590*** | (0.011) |  | 0.589*** | (0.011) |  |  |  |  | 0.592*** | (0.012) |  | 0.590*** | (0.011) |  | 0.590*** | (0.011) |
| Gender - Gender diverse (ref.: male) | 0.673*** | (0.023) |  | 0.672*** | (0.023) |  |  |  |  | 0.680*** | (0.024) |  | 0.672*** | (0.023) |  | 0.672*** | (0.023) |
| Gender - Prefer not to say (ref.: male) | 0.289*** | (0.026) |  | 0.289*** | (0.026) |  |  |  |  | 0.300*** | (0.029) |  | 0.289*** | (0.026) |  | 0.288*** | (0.026) |
| Sexuality - Minority sexual orientation (ref.: heterosexual) | 0.708*** | (0.016) |  | 0.708*** | (0.016) |  | 0.769*** | (0.015) |  | 0.705*** | (0.016) |  | 0.712*** | (0.017) |  | 0.708*** | (0.016) |
| Sexuality - Prefer not to say  (ref.: heterosexual) | 0.266*** | (0.019) |  | 0.266*** | (0.019) |  | 0.275*** | (0.018) |  | 0.266*** | (0.019) |  | 0.279*** | (0.022) |  | 0.266*** | (0.019) |
| FSM (ref.: no FSM) | 0.056*** | (0.012) |  | 0.056*** | (0.012) |  | 0.057*** | (0.012) |  | 0.052*** | (0.012) |  | 0.056*** | (0.012) |  | 0.056*** | (0.013) |
| *Random:* |  |  |  |  |  |  |  |  |  |  |  |  |  |  |  |  |  |
|  | 0.066 | |  | 0.065 | |  | 0.060 | |  | 0.057 | |  | 0.066 | |  | 0.068 | |
| Neighbourhood level (S.D., 95% C.I., S.E.) | (0.051 to 0.086) | |  | (0.050 to 0.085) | |  | (0.044 to 0.082) | |  | (0.040 to 0.080) | |  | (0.051 to 0.085) | |  | (0.053 to 0.087) | |
|  | (0.009) | |  | (0.009) | |  | (0.009) | |  | (0.009) | |  | (0.009) | |  | (0.009) | |
|  | 0.053 | |  |  |  |  |  |  |  |  |  |  |  |  |  |  |  |
| Year group random effect (S.D., 95% C.I., S.E.) | (0.027 to 0.106) | |  |  |  |  |  |  |  |  |  |  |  |  |  |  |  |
|  | (0.019) | |  |  |  |  |  |  |  |  |  |  |  |  |  |  |  |
|  |  |  |  | 0.027 | |  |  |  |  |  |  |  |  |  |  |  |  |
| Ethnicity random effect (S.D., 95% C.I., S.E.) |  |  |  | (0.016 to 0.052) | |  |  |  |  |  |  |  |  |  |  |  |  |
|  |  |  |  | (0.009) | |  |  |  |  |  |  |  |  |  |  |  |  |
|  |  |  |  |  |  |  | 0.063 | |  |  |  |  |  |  |  |  |  |
| Sex random effect (S.D., 95% C.I., S.E.) |  |  |  |  |  |  | (0.040 to 0.098) | |  |  |  |  |  |  |  |  |  |
|  |  |  |  |  |  |  | (0.014) | |  |  |  |  |  |  |  |  |  |
|  |  |  |  |  |  |  |  |  |  | 0.054 | |  |  |  |  |  |  |
| Gender random effect (S.D., 95% C.I., S.E.) |  |  |  |  |  |  |  |  |  | (0.040 to 0.072) | |  |  |  |  |  |  |
|  |  |  |  |  |  |  |  |  |  | (0.008) | |  |  |  |  |  |  |
|  |  |  |  |  |  |  |  |  |  |  |  |  | 0.058 | |  |  |  |
| Sexuality random effect (S.D., 95% C.I., S.E.) |  |  |  |  |  |  |  |  |  |  |  |  | (0.039 to 0.088) | |  |  |  |
|  |  |  |  |  |  |  |  |  |  |  |  |  | (0.012) | |  |  |  |
|  |  |  |  |  |  |  |  |  |  |  |  |  |  |  |  | 0.030 | |
| FSM random effect (S.D., 95% C.I., S.E.) |  |  |  |  |  |  |  |  |  |  |  |  |  |  |  | (0.002 to 0.422) | |
|  |  |  |  |  |  |  |  |  |  |  |  |  |  |  |  | (0.040) | |
|  | 0.895 | |  | 0.895 | |  | 0.893 | |  | 0.894 | |  | 0.894 | |  | 0.895 | |
| Individual level (S.D., 95% C.I., S.E.) | (0.887 to 0.902) | |  | (0.887 to 0.902) | |  | (0.886 to 0.901) | |  | (0.887 to 0.901) | |  | (0.887 to 0.902) | |  | (0.888 to 0.902) | |
|  | (0.004) | |  | (0.004) | |  | (0.004) | |  | (0.004) | |  | (0.004) | |  | (0.004) | |
| S.D. = Standard Deviation; S.E. = Standard Error; C.I. = Confidence Interval; *** p<0.001, ** p<0.01, * p<0.05 | | | | | | | | | | | | | | | | | |
